# Mental Health of Children and Adolescents before and during the COVID-19 Pandemic: How Did the Lockdown Modify Psychiatric Emergencies in Tuscany, Italy?

**DOI:** 10.3390/jcm12124154

**Published:** 2023-06-20

**Authors:** Simone Tavano, Saverio Caini, Idanna Sforzi, Caterina Silvestri, Fabio Voller, Tiziana Pisano

**Affiliations:** 1 Child and Adolescent Psychiatry Unit, Neuroscience Department, Meyer Children’s Hospital IRCCS, Viale Pieraccini 24, 50139 Florence, Italy; simone.tavano@unifi.it; 2 Cancer Risk Factors and Lifestyle Epidemiology Unit, Institute for Cancer Research, Prevention and Clinical Network (ISPRO), 50139 Florence, Italy; s.caini@ispro.toscana.it; 3 Emergency Department, Trauma Center, Meyer Children’s Hospital IRCCS, Viale Pieraccini 24, 50139 Florence, Italy; idanna.sforzi@meyer.it; 4 Regional Health Agency of Tuscany, Via Taddeo Alderotti, 26/N, 50139 Florence, Italy; caterina.silvestri@ars.toscana.it (C.S.); fabio.voller@ars.toscana.it (F.V.)

**Keywords:** children, adolescents, mental health, psychiatric emergency, lockdown, COVID-19 pandemic

## Abstract

The COVID-19 pandemic has deeply impacted several aspects of the lives of children and adolescents. We analyzed the trends of psychiatric disorders in the emergency room. The analysis comprised the pre-pandemic (2018–2019) and the pandemic years (2020–2021). We conducted a retrospective observational epidemiological study that compared admissions during the two periods on a sample of 1311 patients aged between 4 and 18, focusing on new admissions vs. relapse, demographic variables, lockdown severity, presentation of psychiatric symptoms, diagnosis, severity, and outcome. Over the two-year pandemic period, we observed a 33% decrease in admissions to the emergency room for non-psychiatric disorders and a 200% increase in admissions for psychiatric emergencies. This increase is concentrated in periods with fewer restrictions and in the second year of the pandemic. We also observed a greater impact of psychiatric disorders on female patients, a greater severity of psychiatric disorders, a change in diagnoses associated with the presentation of symptoms, and an increase in hospitalizations. The children’s psychiatric emergency service faced an “emergency within the emergency”. In the future, it will be necessary to continue the follow-up of these patients, strengthen the field of study of gender psychiatry, and intensify our efforts towards prevention.

## 1. Introduction

In 2010, the World Health Organization (WHO) projected neuropsychiatric disorders to be one of the five leading causes of morbidity, mortality, and disability by 2020 [1]. A steady decrease in the availability of psychiatric services was, nonetheless, observed over the years in outpatient and inpatient care for minors and adults. This led patients, many with psychiatric symptoms, to skip the first level of care, i.e., territorial services, referring directly to the emergency room regardless of the symptoms’ severity [2], resulting in an increase in new access to the relevant emergency room. In fact, the hospitalization rate after going to the emergency room is 30%, i.e., only one patient out of three is hospitalized [3].

The WHO, however, could not forecast the COVID-19 pandemic. Italy was the earliest European country to be involved in the pandemic and contained infection by taking timely measures [4]. Some study groups raised concerns about the harmful effects on mental health, in both adults and children, of these containment measures already in 2020 [5,6]. The negative correlation between pandemic or epidemic events, lockdown, and mental health has already been studied by disaster psychiatry during the SARS-CoV-1 [7] and MERS-CoV epidemics [8]. After the 2008 SARS epidemic in Beijing (China), for example, some authors highlight, among people subjected to quarantine, an increase in depressive symptoms even 3 years after the epidemic [7]. During the 2015 MERS outbreak in Korea, 80.2% of the population reported fear of being infected and 46% reported emotional distress. The risk factors associated with fear include attending crowded places or using public transport, the perception of poor protection by institutions, and impotence in the face of situations that cannot be controlled [8]. Fear or anxiety and anger are emotions commonly expressed in times of uncertainty. For this reason, the Italian Ministry of Health published a guideline document for emergency management in the psychiatric field, having already considered the real mental health risk, in April 2020. High levels of stress in adults and children, and mental health conditions worsening were reported by some studies during spring 2020 [9,10], also in Italy [11]. In the United States, for example, an increase in suicidal ideation rates and suicide attempts was reported during spring and summer 2020 [12].

The examination of admissions to the emergency room for psychiatric symptoms can thus offer the opportunity to quickly assess the trend of psychiatric disorders in a specific area, in this case the emergency room of the Meyer Children’s Hospital—IRCCS for Tuscany, Italy. The population aged 4–18 years old is composed of 479,071 subjects in Tuscany [13], and this is the only regional pediatric emergency room where psychiatric emergencies of developmental age are hospitalized.

The purpose of this study is to carry out a complete trend analysis of all psychiatric patients of developmental age admitted to the emergency room in the pre-pandemic period (2018–2019) and during the initial two waves of the COVID-19 pandemic (2020–2021).

The primary objective is to identify and compare modifications in volume, demographic variables, type of psychopathology, and severity, within both new diagnoses and relapses, during the two two-year time frames in question. The secondary purpose is to formulate hypotheses about the causes of this trend.

## 2. Materials and Methods

### 2.1. Study Population

The study population includes all children between 4 and 18 years of age who were admitted consecutively to the Meyer Children’s Hospital—IRCCS emergency room due to psychiatric symptoms in the two-year pre-pandemic time frame (2018–2019) and during the first two years of the COVID-19 pandemic (2020–2021).

### 2.2. Data Collection

Data were extracted from the electronic health records of the Meyer Children’s Hospital—IRCCS emergency room and psychiatry department, then anonymized by assigning a numeric code to each patient and collected into a database.

The following data were collected for each patient:

- Date of birth (day, month, year).
- Sex (male, female).
- Emergency room admission date (day, month, year).
- Psychiatric symptoms at admission (affective, anxiety, eating disorder, psychotic, psychomotor agitations, self-harm, suicide attempt, suicidal ideation, and somatic symptoms).
- New admissions versus relapse.
- None or previous psychiatric diagnosis according to the Diagnostic and Statistical Manual of Mental Disorder (DSM-5-TR) criteria.
- Outcome after psychiatry specialist evaluation (home discharge versus hospitalization) was used as the measure of clinical severity.

### 2.3. Lockdown Severity

The lockdown severity (LS) was established and modified over time according to the course of the pandemic by the Italian Government with the purpose of containing the virus spread. We established five LS increasing levels, from LS-1 to LS-5, after examining the decrees issued by the government [14]. In detail:

- LS-1: no restrictions on social contacts, work activities, or travel, routine health care guaranteed, schools fully open.
- LS-2: restaurants open only during daytime, recreational activities not allowed, travel within the region is unrestricted, travel between regions not allowed, routine health care guaranteed, schools open only for students up to 14 years.
- LS-3: restaurants are closed, recreational activities not allowed, movement allowed only within the city of residence, social contacts limited to two people per day, routine health care guaranteed, secondary school students attend classes through distance learning, schools open only for students up to 11 years.
- LS-4: restaurants and non-essential commercial activities are closed, leisure activities not allowed, travel not allowed, 24 h curfew, home exit allowed only for essential activities, routine health care guaranteed, most schools are closed or use distance learning.
- LS-5: same as the previous level, plus all routine health is suspended because medical services have been limited to essential needs and urgent care, all schools are closed or use only distance learning.

This classification is like the colors used by the Italian Government for the region classification according to local pandemic conditions: white (LS-1), yellow (LS-2), orange (LS-3), and red (LS-4 and LS-5). Each month has been, hence, associated to a single level of lockdown severity from LS-1 to LS-5 to facilitate statistical data analysis. This classification is valid for Tuscany only (Table 1).

### 2.4. Data Analysis

Data were analyzed using the statistical software STATA, version 17. The distribution of the study population’s variables was analyzed using averages and medians for continuous variables and percentages for categorical variables. We also fitted multivariable logistic regression models in order to identify the factors associated with the odds of being hospitalized, separately among subjects presenting with the different symptoms.

## 3. Results

### 3.1. Total and Psychiatric Clinical Presentations Admissions

We recorded a total of 143,909 admissions for any medical cause at the Meyer’s Children Hospital—IRCCS emergency room in the study period (2018–2021), and subdivided them as follows: 42,923 admissions in 2018, 43,111 in 2019, 24,906 in 2020, and 32,969 in 2021. There were 1311 admissions for psychiatric clinical presentations divided as follows: 226 admissions in 2018, 290 in 2019, 250 in 2020, and 537 in 2021. The admission rates for psychiatric disorders represented 0.53% in 2018, 0.67% in 2019, 1.04% in 2020, and 1.63% in 2021 of total admissions. Further division of psychiatric clinical presentations in new admissions and relapses showed 79 new admissions and 147 relapses in 2018, 73 new admissions and 217 relapses in 2019, 76 new admissions and 182 relapses in 2020, and 156 new admissions and 381 relapses in 2021. The new admissions and relapses, as a percentage of total admissions for psychiatric clinical presentations, were, respectively, 35% and 65% in 2018, 25.2% and 74.8% in 2019, 29.5% and 70.5% in 2020, and 29.1% and 70.9% in 2021 (Table 2).

### 3.2. Demographic Variables of Admissions for Psychiatric Clinical Presentations

Biological sex and age are the demographic variables considered regarding the admission for psychiatric clinical presentation. Males and females, and their values as a percentage of total admissions were as follows: 105 males (46.5%) and 121 females (53.5%) in 2018, 140 males (48.3%) and 150 females (51.7%) in 2019, 93 males (36%) and 165 females (64%) in 2020, and 169 males (31.5%) and 368 females (68.5%) in 2021.

The average age at admission for psychiatric clinical presentation was as follows: 13.9 in 2018, 14.6 in 2019, 14.4 in 2020, and 14.5 in 2021.

### 3.3. Monthly Average Admissions of Psychiatric Clinical Presentations

The pre-pandemic period runs from January 2018 to February 2020, for a total of 26 months, with an average of 21.7 psychiatric clinical presentations admissions per month, consisting of 6.3 new admissions and 15.4 relapses.

The pandemic period runs from March 2020 to December 2021, for a total of 22 months, further divided according to the lockdown severity (LS) levels as follows: 5 months LS-1, 7 months LS-2, 2 months LS-3, 2 months LS-4, and 6 months LS-5. Details are shown in Table 3.

The monthly average admissions of psychiatric clinical presentations in the five periods were as follows: 43.6 admissions (11.2 new and 32.4 relapses) in LS-1, 37.7 admissions (12.4 new and 25.3 relapses) in LS-2, 30.5 admissions (8 new and 22.5 relapses) in LS-3, 31 admissions (8 new and 23 relapses) in LS-4, and 23.7 admissions (7 new and 16 relapses) in LS-5.

### 3.4. Admission Symptoms for Psychiatric Clinical Presentations in All Patients and Related to the Biological Sex

**Figure 1:**
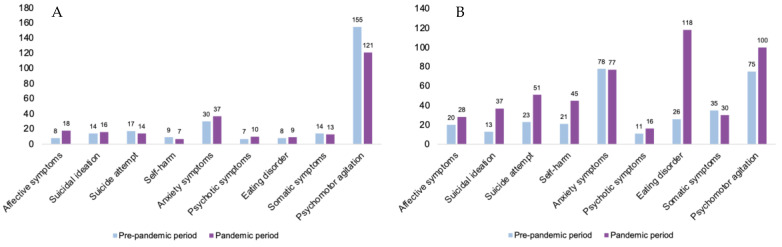
Admission symptoms for psychiatric clinical presentations in females (**A**) and males (**B**) in the pre-pandemic period (January 2018–February 2020) and the pandemic period (March 2020–December 2021).

During the pandemic period, we saw an increase in suicide attempts, eating disorders, self-harm, affective symptoms, and suicidal ideation. The symptoms of psychiatric admission among all patients (male and female) in the pre-pandemic and the pandemic period were highest or lowest by frequency as shown in Table 4. If we consider the biological sex, psychomotor agitation as a presenting symptom is observed in both sexes, while in females there is an increase in eating disorders, suicidal attempts, suicidal ideation, and self-harm, whereas in males there is a prevalence in affective symptoms (Figure 1).

### 3.5. Symptoms and Diagnosis of Psychiatric Clinical Presentations Admissions

We analyzed the underlying psychiatric diagnosis in the pre-pandemic and pandemic period starting from the symptoms with which patients admitted to the emergency room (Table 5).

### 3.6. Admissions Outcome of Psychiatric Clinical Presentations

In multivariable logistic regression models, an older age was associated with increased odds of hospitalization among patients with affective symptoms (by +1 year of age: OR 1.64, 95% CI 1.11–2.42, *p*-value 0.013) as well as psychomotor agitation (OR 1.18, 95% CI 1.08–1.30, *p*-value < 0.001), while male sex was associated with reduced odds of hospitalization among patients with somatic symptoms. Patients with anxiety symptoms were more likely to be hospitalized in cases where the access was due to a relapse of the illness, while those with psychotic symptoms were less likely. We identified no determinants of hospitalization among patients characterized by suicidal ideation, suicidal attempt, or self-harm. Having already accessed the emergency room in the past was associated with increased odds of hospitalization among patients with anxiety symptoms and reduced odds among those with eating disorders or psychotic symptoms. Finally, the type of ongoing treatment had an impact on the chance of being hospitalized among patients with anxiety symptoms, eating disorders, and somatic symptoms (Table 6).

**Figure 2:**
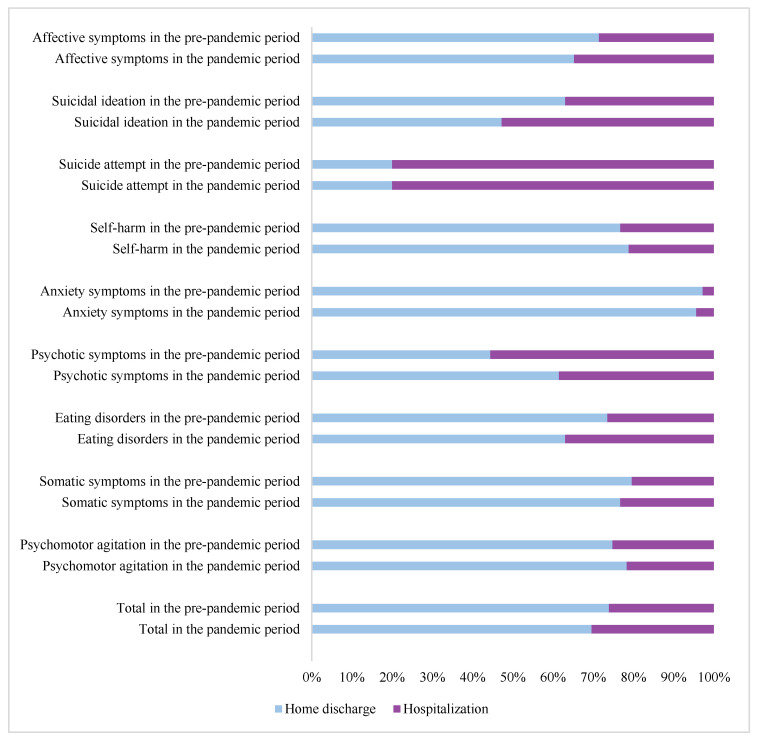
Admission outcome of psychiatric clinical presentations in the pre-pandemic period (January 2018–February 2020) and the pandemic period (March 2020–December 2021).

In this study, we assessed the admission severity for psychiatric clinical presentations considering the intensity of care decided at the end of the specialist’s assessment in the emergency room. In detail, home discharge was considered a positive outcome, while being hospitalized was a negative one. In particular, we have documented an increase in hospitalizations during the pandemic and especially for eating disorders, suicidal ideation, and affective symptoms. There was no difference in suicide attempts; in fact, the hospitalization rate was 80% before the pandemic as well as during the pandemic (Figure 2).

## 4. Discussion

The direct effects of the COVID-19 pandemic and the measures implemented to contain the virus diffusion changed everyone’s life [15]. These aspects, combined with personal vulnerability of younger people and their parents, social and environmental factors, interpersonal dynamics, and system failure, provided the substrate for the so-called “perfect storm” [16].

Children and adolescents are less affected by COVID-19 infection and its complications, but they have a higher risk of developing a psychiatric disorder due to their young age. The total number of admissions, in fact, decreased by 33% in the Meyer Children’s Hospital—IRCCS emergency room during the pandemic. However, the admissions to psychiatric services increased by 54%, from 0.53% of total admissions in 2018 to 1.63% in 2021, a 200% increase. Our findings were similar to other studies [17,18,19]. Nevertheless, two other studies reported a decline in psychiatric ED referrals during the pandemic [20,21]; however, both were limited to a short-term period of analysis near the lockdown. We observed an increase in admissions for psychiatric clinical presentations in the second year of the pandemic, i.e., 2021, but not in 2020, while the admissions decreased in March 2020 compared with March 2019. A similar finding was also described in a multicenter study conducted in several Italian hospital pediatric emergency rooms [22]. COVID-19 restrictions may have had an initial protective effect on mental health, reducing school stress, social exposures, fear of infection, and hypnic deprivation [23]. Indeed, some children reported feeling better during the early stages of lockdown [6]. The relationship between stress and mental health can explain this hypothesis. The activation of physiological mechanisms can overcome acute stress, which is less related to mental disorders, unlike chronic or prolonged stress [24].

Among our 1311 patients, the ratio of new admissions to relapses was unchanged during the pre-pandemic and pandemic period, reaching approximately 70%. This data confirms that the patients with a previous psychiatric diagnosis were the most fragile during the whole study period. There was a discontinuity of treatment, resulting in worsening of psychopathological clinical presentation, during the pandemic, as demonstrated in this study.

The gender ratio is similar in the pre-pandemic period, while there is a large increase in female subjects, which represented 64% in 2020 and 68.5% in 2021. The different impact of the pandemic on females has been studied in several works with similar data in the adult population [25]. It is difficult to explain the reason for this difference between the two sexes. This aspect will need to be explored in the field of gender psychiatry.

It has been demonstrated that lockdown and loneliness can also affect mental health, increasing the risk of psychiatric disorders [26]. The lockdown severity was modified over time, through decrees, according to the pandemic trend, throughout the pandemic period. Five levels of increasing lockdown severity, from LS-1 to LS-5, have been established considering some crucial aspects, such as restrictions on social contacts, travel, obligation to wear a mask in public, the guaranteed level of health care, and school or work manner of participation. The pre-pandemic period runs from January 2018 to February 2020 (26 months), while the pandemic period runs from March 2020 to December 2021 (22 months). We assigned a single level of lockdown severity from LS-1 to LS-5 to each month, with the aim of observing the variations in the admissions to emergency rooms in relation to the restrictions. We would like to emphasize that primary health care is present from LS-1 to LS-4, and it is suspended in LS-5. Patients in need of health care were, therefore, forced to go to the emergency room. It seemed rational to expect an increase in the emergency room admissions in the LS-5 period, but the opposite happened. For both the admissions for psychiatric clinical presentations and non-psychiatric ones there was an increase in the monthly average admissions in the LS-1 period and a gradual decrease in the other periods, with the minimum in the LS-5 period. The causes of the increase in the LS-1 period are complex to evaluate. They could be related to new social, educational, and work exposure, an easier perception of admission to treatment evaluation at hospital centers, or real or perceived emergency by the patient or families. Fear of infection could also have caused the delayed access to care, with a consequent worsening of psychopathological clinical presentations during the LS-5 period.

On the other hand, some authors, have demonstrated the protective value of staying at home [22,27], as happened especially in the LS-5 period.

It is possible to hypothesize that the restrictions and forced cohabitation with family imposed at the LS-5 level had a negative impact on mental health, resulting in increased admissions during the period of low lockdown intensity, as observed in our study. It is known in psychiatry that non-exposure to stressful situations initially produces a benefit but, subsequently results in a reduction in skills and it increases a subject’s salience of feared or avoided situations. The return to the complexity of a new daily routine after several months could explain this trend in admissions.

Regarding the seasonal variables, usually psychiatric admissions have a peak in spring and autumn with a plateau during the summer. In our case, it is difficult to evaluate the seasonality of admissions because of the lockdown bias and so it is impossible to compare the admissions per month between 2020 and 2021. The only months with the same level of lockdown severity in the two years of the pandemic that can be compared are April (LS-5), with 27 admissions in 2020 and 47 in 2021, and June (LS-2) with 16 admissions in 2020 and 49 in 2021. These data confirm that there was an increase in the number of admissions in 2021 compared to 2020, being the same in terms of lockdown severity.

We observed, in males and females of our sample, an increase in emergency room admissions due to eating symptoms, affective symptoms, suicidal ideation, attempted suicide, and self-harm during the pandemic period. Psychotic symptoms, anxiety, and somatic symptoms admissions essentially remained unchanged, while we observed a slight decrease in psychomotor agitation admissions.

Comparing the hospitalizations in the two periods and considering the variable biological sex, there was a greater increase in the frequency of affective and anxiety symptoms in males, while there was a particular increase in affective symptoms, suicidal ideation, suicidal attempts, self-harm, eating disorder symptoms, and psychomotor agitation in females.

We observed that the symptoms of clinical presentations in patients with a previous diagnosis are different in the pandemic and in the pre-pandemic periods. These observations may be useful to explain the causes of the increase in some symptoms and should be considered by the clinician during the process of the specialist’s assessment in first aid over the pandemic era.

We noted a particular increase in feeding and eating disorders in the pandemic period. These disorders have a psychopathological core related to low personal self-esteem, clinical perfectionism, brooding, and a pervasive sense of responsibility, which mainly affects females [28]. Individuals tend to relate their performance value to various domains of life (e.g., interpersonal relationships, school, work, sports, intellectual abilities), for example, people with feeding and eating disorders relate their personal value to the control upon weight, nutrition, and body shape. This maladaptive scheme grants the subject strict control over an aspect of life with consequent feelings of safety and contentment [29]. Many female patients in our study reported that they started a “do-it-yourself” diet and exercise to get back in shape, with a consequent progressive increase in interest in calories, weight, and body shape during the uncertainty of lockdown. A sedentary lifestyle and reduced physical activity, combined with an unbalanced diet and the insecurity experienced during lockdown, could have been a trigger factor for the onset of eating and nutrition disorders. The use of smartphones and the internet during the pandemic may explain the tendency to compare, resulting in increased self-criticism and worsened eating disorder symptoms [30]. Eating disorders are often a comorbidity with mood disorders and, sometimes, there is a mutual relationship. It is reasonable to hypothesize that isolation, altered daily routine and hypnic pattern, and interpersonal dynamics with parents deriving from prolonged cohabitation, may have caused the onset of affective symptoms and the worsening of eating disorders at the developmental age. In line with our study, the Edit 2022 Tuscany survey [31], carried out on the adolescent population showed that the data on the level of perceived discomfort largely concerned females and this could represent an additional risk factor for eating disorders [32]. Our study data suggest an increase in affective symptoms, suicidal ideation, suicide attempts, self-harm, and anxiety symptoms in subjects with feeding and eating disorders, as some authors have previously described [33]. On the other hand, we did not observe an increase in access for eating symptoms in the male sex. More frequently males present vigorexia instead. It is, therefore, likely that many male subjects have also started a “do-it-yourself” diet or exercise in pandemic times without reaching a level of severity that requires an assessment at an emergency room.

We also noted an increase in the number and frequency of admissions for affective symptoms, suicidal behaviors, and self-harm, especially in women, during the pandemic. The possible explanations at the base of the increase in such disturbances could be the alteration of daily routines and of circadian rhythms, continuous alternation between closing and reopening of the schools, reduction in socialization with peers in person and of daily physical activity, fear of infection, socio-economic difficulties, and a possible increase in family conflict. An epidemiological study conducted at the IRCCS “Giannina Gaslini” in Genoa, identified the psychological impact of the COVID-19 pandemic on Italian families from March to April 2020. The results show that 65% of children under the age of six showed an increase in irritability, sleep disorders, and anxiety disorders, while 71% of children over the age of six had behavioral regression and a significant change in the sleep–wake rhythm. Adolescents, on the other hand, exhibited increased emotional instability with increased irritability. The study also noted that the severity of children’s and adolescents’ symptoms would appear to be related to the degree of discomfort which parents experienced during lockdown [11]. Confirming the greater vulnerability of adolescents, in our study we also observed that increased age carries with it a greater risk of hospitalization.

Another important factor could be the reduction or discontinuation of sports activity. One study found that children who engaged regularly in physical activity during the pandemic had generally more harmonious development, better pro-social abilities, and fewer mood disorders, hyperactivity, and inattention [34]. The fear of infection appears to be another factor directly related to mental health. A study examined the negative effects in the pediatric population living in areas with a high rate of infection, finding higher levels of mood disorders, and relational and behavioral difficulties [35]. Many Tuscan districts were classified as red areas for long periods, and this may have contributed to the increase in, and aggravation of, psychiatric symptoms in the population during the pandemic waves. Another study noted that primary school children showed increased concern about their life and health during pandemic waves [36]. An Italian study, carried out with a questionnaire in secondary schools, demonstrated that the most serious psychological consequences were recorded among adolescents who had a relative or an acquaintance with a COVID-19 infection [37]. The overwhelming and inadequate spread of news about COVID-19 by the media also seems to have had an impact on mental health. In a large study conducted among primary school children, it was observed that the subjects who spent the most time listening to the news about COVID-19, without the mediation of an adult, reported higher levels of anxiety, mood disorders, and symptoms associated with post-traumatic stress disorder [38]. It is difficult to separate the psychological well-being of children from that of their families, and thus, it is possible to suppose the adults’ well-being inevitably affected their parenting ability. One study examined the correlation between parents’ and children’s psychological well-being, finding a correlation with work difficulties and reduced parental income [39]. An American study showed that 26.9% of the parents reported a deterioration in their mental health after the onset of the pandemic. This worsening has been seen in mothers, unmarried parents, and families with younger children [40]. Studies conducted during the COVID-19 pandemic also found that parents’ depression, job loss, and previous mistreatment predicted higher rates of mistreatment of children aged 4 to 10 years old [41].

We observed an increase in the percentage and in the absolute number of hospitalizations regarding the severity of the clinical presentations during the pandemic period (30.4% vs. 26.1%). This could be explained by a possible increase in the severity of some psychopathological clinical presentations in the pandemic period. Delayed access to care, due to fear of infection and discontinuity of primary care services, could have caused a greater severity of symptoms. There was no difference in suicide attempts; in fact, the hospitalization rate was 80% before the pandemic as well as during the pandemic, confirming the need for a protected environment in the face of suicidal ideation and suicide attempts.

The main strengths of this study are the size and uniformity of the sample, time of observation, and use of a scale of assessment for lockdown severity. The main weakness lies in the absence of epidemiological data regarding the total number of psychiatric patients in Tuscany during the study period. These data could not be found due to the absence of a regional register of psychiatric cases. The presence of these data would have allowed us to highlight a variation in the incidence and prevalence of psychiatric pathology over the study period. Our data refer to only one region, Tuscany, Italy, and therefore, it is not possible to draw general conclusions on the trend of psychiatric emergencies in Italy. In the future it would be desirable to extend the analysis to Italy as a whole. In any case, this analysis would still be complex to perform because of the different levels of lockdown in the Italian regions during the pandemic.

## 5. Conclusions

We observed an increase in the number and percentage of emergency room admissions for psychiatric clinical presentations, in comparison to a decrease in other admissions, during the pandemic period. This increase is mainly concentrated in the less restricted time frames and in the second year of the pandemic, i.e., 2021. This, as stated by Cancilliere and Donise [42], could be related to a lack of outpatient mental health services that aim to prevent acute mental health crises.

There is also a change in semeiology and an increase in admissions for the following symptoms: eating disorder symptoms, affective symptoms, suicidal ideation, suicide attempts, and self-harm, with a greater involvement of females and an increase in severity of clinical presentations, percentage, and number of admissions. There are no changes in the ratio of new admissions vs. relapses.

It seems useful to further investigate the reasons for the increase in each symptom and to continue the follow-up of these patients to understand when the pandemic effects on mental health will end. We will also need to strengthen the field of study of gender psychiatry, to create a unified regional register of psychiatric disorders, and to intensify our efforts toward prevention.

However, we must not underestimate the social aspects that may have contributed to the worsening of the psychiatric symptoms linked to the COVID-19 pandemic. Some studies have highlighted the need to pay attention to the aspects linked to communication of information which, during the pandemic, should be monitored and directed towards avoiding fake news to maintain social comfort as well as to fight COVID-19 infection [15,43].

## Figures and Tables

**Table 1:** Day, month, and year of the decrees and lockdown severity (LS) in Tuscany region, Italy.

Decrees and Levels (2020)	Decrees and Levels (2021)
-	14 January (LS-3)
-	12, 23 February (LS-4)
1, 4, 8, 11, 20, 22, 25 March 2020 (LS-5)	2 March 2021 (LS-4)
10, 23, 26 April 2020 (LS-5)	22 April 2021 (LS5)
17 May 2020 (LS-5)	18 May 2021 (LS-2)
11 June 2020 (LS-2)	No decrees (LS-2)
No decrees (LS-2)	23 July 2021 (LS-1)
No decrees (LS-2)	6 August 2021 (LS-1)
7 September 2020 (LS-2)	No decrees (LS-1)
13, 18, 24 October 2020 (LS-3)	8 October 2021 (LS-1)
3 November 2020 (LS-5)	No decrees (LS-1)
2, 3 December 2020 (LS-5)	24 December 2021 (LS-2)

Source: Official Gazette of the Italian Republic, 2020–2021.

**Table 2:** Total admissions, new, and relapse admissions for mental health in the emergency room in the study period (2018–2021).

Year	Total Admissions in ER	MH Admissions (N and Percentage of Total)	New MH (N and Percentage of Total)	Relapses MH (N and Percentage of Total)
2018–2021	143,909	1311/143,909 (0.91%)	384/1311 (29.3%)	927 (70.7%)
2018	42,923	226/42,923 (0.53%)	79/226 (35%)	147 (65%)
2019	43,111	290/4311 (0.67%)	73/290 (25.2%)	217 (74.8%)
2020	24,906	258/24,906 (1.04%)	76/258 (29.5%)	182 (70.5%)
2021	32,969	537/32,969 (1.63%)	156/537 (29.1%)	381 (70.9%)

Legend: ER = emergency room; MH = mental health; N = number.

**Table 3:** Monthly average admissions of psychiatric clinical presentations in the pandemic period (March 2020–December 2021).

Pandemic Period (March 2020–December 2021)				
/	2020		2021	
Month	Levels	Admissions	Levels	Admissions
January	/	/	LS-3	39
February	/	/	LS-4	28
March	LS-5	9	LS-4	34
April	LS-5	17	LS-5	47
May	LS-5	20	LS-2	64
June	LS-2	16	LS-2	49
July	LS-2	21	LS-1	36
August	LS-2	21	LS-1	42
September	LS-2	35	LS-1	42
October	LS-3	22	LS-1	37
November	LS-5	20	LS-1	61
December	LS-5	29	LS-2	58

**Table 4:** Admission symptoms for psychiatric clinical presentations in the sample in the pre-pandemic period (January 2018–February 2020) and the pandemic period (March 2020–December 2021).

Admission Symptoms for Psychiatric Clinical Presentations	Pre-Pandemic Period (January 2018–February 2020)		Pandemic Period (March 2020–December 2021)	
Psychomotor agitation	230	40.8%	221	29.5%
Anxiety symptoms	108	19.1%	114	15.3%
Somatic symptoms	49	8.7%	43	5.7%
Suicide attempt	40	7.1%	65	8.7%
Eating disorder	34	6.1%	127	17%
Self-harm	30	5.3%	52	7.1%
Affective symptoms	28	4.9%	46	6.1%
Suicidal ideation	27	4.8%	53	7.1%
Psychotic symptoms	18	3.2%	26	3.5%

**Table 5:** The table shows the three most frequent psychiatric diagnoses associated with the admissions symptoms in the emergency room both in the pre-pandemic period (January 2018–February 2020) and in the pandemic period (March 2020–December 2021).

Admission Symptoms for Psychiatric Clinical Presentations	Pre-Pandemic Period (January 2018–February 2020)			Admission Symptoms for Psychiatric Clinical Presentations	Pandemic Period (March 2020–December 2021)		
Affective symptoms	DD (52.2%)	FEDs (8.7%)	DIC (8.7%)	Affective symptoms	DD (72.2%)	DIC (8.3%)	FEDs (8.3%)
Suicidal ideation	DD (65%)	ID (15%)	Bipolar disorders (10%)	Suicidal ideation	DD (61%)	FEDs (14.6%)	Bipolar disorders (7.3%)
Suicide attempt	DD (53.6%)	FEDs (17.9%)	Anxiety disorders (7.1%)	Suicide attempt	DD (66.7%)	FEDs (16.7%)	Schizophrenia (4.2%)
Self-harm	DD (34.8%)	FEDs (17.4%)	Bipolar disorders (8.7%)	Self-harm	DD (45.7%)	FEDs (17.1%)	Anxiety disorders (8.6%)
Anxiety symptoms	Anxiety disorders (53.1%)	DD (12.2%)	None (10.02%)	Anxiety symptoms	Anxiety disorders (62.1%)	DD (17.2%)	None (10.3%)
Psychotic symptoms	Schizophrenia (50%)	ID (12.5%)	Depressive disorders (12.5%)	Psychotic symptoms	Schizophrenia (30%)	DD (20%)	DIC (10%)
Eating disorder	FEDs (72.7%)	None (13.6%)	Intellectual disabilities (9.1%)	Eating disorder	FEDs (88.8%)	DD (3.7%)	Anxiety disorders (1.2%)
Somatic symptoms	Somatic disorders (60%)	ID (10%)	ADHD (10%)	Somatic symptoms	Somatic disorders (33.3%)	None (18.5%)	DD (14.8%)
Psychomotor agitation	DD (33.2%)	ID (15.8%)	ASD (10.2%)	Psychomotor agitation	DIC (29.7%)	ID (17.1%)	ADHD (12.6%)

Legend: FEDs: feeding and eating disorders; ADHD: attention deficit and hyperactivity disorder; ASD: autism spectrum disorder; DD: depressive disorder; DIC: disruptive, impulse control, and conduct disorders; ID: intellectual disabilities.

**Table 6:** This table reports the factors associated (with *p*-value < 0.100) with the risk of hospitalization, separately for the different types of symptoms at presentation.

	OR	95% CI	*p*-Value
*Psychomotor agitation*			
Age (+1 year)	1.18	1.08–1.30	<0.001
*Anxiety symptoms*			
Previous access			
No	1.00		
Yes, and this is a relapse	10.28	1.01–105.12	0.049
Yes, but this is not a relapse	-	-	-
Treatment: educational (yes vs. no)	83.27	4.15–1670.55	0.004
*Eating disorder*			
Previous access			
No	100		
Yes, and this is a relapse	0.33	0.10–1.11	0.074
Yes, but this is not a relapse	0.10	0.01–1.22	0.071
Treatment: psychotherapy (yes vs. no)	0.31	0.14–0.072	0.006
*Somatic symptoms*			
Sex			
Female	1.00		
Male	0.08	0.01–0.72	0.024
Treatment: drugs (yes vs. no)	0.18	0.04–0.77	0.021
Treatment: psychotherapy (yes vs. no)	9.92	2.54–38.72	0.001
*Affective symptoms*			
Age (+1 year)	1.64	1.11–2.42	0.013
*Psychotic symptoms*			
Previous access			
No	1.00		
Yes, and this is a relapse	0.20	0.05–0.79	0.022
Yes, but this is not a relapse	0.30	0.02–2.42	0.260

## Data Availability

The dataset generated during the current study are available from the corresponding author.
